# Supplementary material for: A Likelihood Approach for Real-Time Calibration of Stochastic Compartmental Epidemic Models
Source: PLoS Comput Biol. 2017 Jan 17;13(1):e1005257. doi: 10.1371/journal.pcbi.1005257 (PMC5240920; doi:10.1371/journal.pcbi.1005257)
Supplement: S1 File — (TAR.GZ) [file pcbi.1005257.s014.tar.gz › HSPH_Online-SI-Revision/EnKF07/n10-mild/n10-mild_EnKF2_table0.pdf]

| | | | | | | | | | | | | | | | | | | | | | | | | | | | | | | |
|---|---|---|---|---|---|---|---|---|---|---|---|---|---|---|---|---|---|---|---|---|---|---|---|---|---|---|---|---|---|---|
| 1 | newly 3 weeks spec | 176. | 260.33 | {40., 480.} | 95.3125 | 0.31728 | newly 3 weeks cumul | 530. | 2259.77 | {561., 8269.} | 334.594 | 0.398037 | newly 5 weeks spec | 135. | 91.7 | {2., 124.} | 88.7111 | 0.780332 | newly 5 weeks cumul | 825. | 2494.59 | {636., 8639.} | 217.276 | 0.320165 | newly 50 weeks cumul | 2160. | 2713.22 | {660., 8641.} | 105.514 | 0.41529 |
| 2 | newly 3 weeks spec | 316. | 100.24 | {0., 108.} | 105.31 | 1.955 | newly 3 weeks cumul | 1222. | 983.27 | {61., 2110.} | 113.554 | 0.73698 | newly 5 weeks spec | 183. | 42.37 | {0., 60.} | 99.0765 | 1.899 | newly 5 weeks cumul | 1613. | 1090.94 | {65., 2110.} | 107.262 | 0.776207 | newly 55 weeks cumul | 2172. | 1177.09 | {72., 2110.} | 99.4894 | 0.827247 |
| 3 | newly 3 weeks spec | 434. | 85.1 | {12., 145.} | 83.5714 | 1.01856 | newly 3 weeks cumul | 1373. | 499.49 | {63., 737.} | 79.4588 | 0.74248 | newly 5 weeks spec | 309. | 39.82 | {2., 63.} | 91.3333 | 1.42874 | newly 5 weeks cumul | 2076. | 594.41 | {69., 877.} | 81.0072 | 0.834052 | newly 58 weeks cumul | 2924. | 692.23 | {89., 1161.} | 81.9402 | 0.909046 |
| 4 | newly 3 weeks spec | 417. | 161.45 | {5., 391.} | 86.952 | 0.964882 | newly 3 weeks cumul | 1378. | 692.11 | {90., 1554.} | 81.6103 | 0.687008 | newly 5 weeks spec | 196. | 72.29 | {0., 143.} | 85.5255 | 1.16957 | newly 5 weeks cumul | 1896. | 869.55 | {110., 2035.} | 81.4836 | 0.719532 | newly 53 weeks cumul | 2527. | 1077.57 | {118., 2369.} | 80.5038 | 0.746477 |
| 5 | newly 3 weeks spec | 111. | 354.6 | {103., 951.} | 232.541 | 0.416953 | newly 3 weeks cumul | 344. | 1894.09 | {1009., 3736.} | 451.259 | 0.646308 | newly 5 weeks spec | 94. | 176.21 | {9., 344.} | 155.862 | 0.649408 | newly 5 weeks cumul | 547. | 2310.96 | {1313., 4736.} | 323.218 | 0.531765 | newly 33 weeks cumul | 2021. | 2813.72 | {1426., 6802.} | 68.5908 | 0.184529 |
| 6 | newly 3 weeks spec | 331. | 210.88 | {0., 729.} | 113.855 | 1.39726 | newly 3 weeks cumul | 1004. | 1586.47 | {35., 7753.} | 178.328 | 0.792507 | newly 5 weeks spec | 169. | 41.94 | {0., 50.} | 100.083 | 1.96174 | newly 5 weeks cumul | 1390. | 1714.78 | {38., 7914.} | 152.309 | 0.856749 | newly 59 weeks cumul | 2050. | 1799.17 | {43., 7922.} | 124.264 | 0.937833 |
| 7 | newly 3 weeks spec | 277. | 188.74 | {1., 110.} | 124.874 | 1.19676 | newly 3 weeks cumul | 873. | 1237.9 | {87., 3457.} | 152.011 | 0.620526 | newly 5 weeks spec | 186. | 75.1 | {0., 81.} | 108.731 | 1.66872 | newly 5 weeks cumul | 1277. | 1435.8 | {106., 5077.} | 136.924 | 0.696623 | newly 55 weeks cumul | 1892. | 1563.86 | {122., 5077.} | 118.686 | 0.786677 |
| 8 | newly 3 weeks spec | 269. | 160.96 | {2., 366.} | 80.1115 | 0.869167 | newly 3 weeks cumul | 743. | 2124.78 | {492., 6688.} | 210.767 | 0.382252 | newly 5 weeks spec | 224. | 21.55 | {0., 52.} | 91.4955 | 1.89505 | newly 5 weeks cumul | 1216. | 2201.97 | {499., 6899.} | 140.927 | 0.418031 | newly 50 weeks cumul | 3026. | 2219.57 | {499., 6917.} | 77.535 | 0.545232 |
| 9 | newly 3 weeks spec | 476. | 127.52 | {49., 174.} | 87.3908 | 0.790496 | newly 3 weeks cumul | 1520. | 526.05 | {232., 628.} | 82.4743 | 0.650607 | newly 5 weeks spec | 288. | 72.15 | {15., 104.} | 86.8021 | 0.885242 | newly 5 weeks cumul | 2165. | 692.43 | {299., 914.} | 83.691 | 0.68793 | newly 54 weeks cumul | 2749. | 991.53 | {382., 1353.} | 83.5638 | 0.679001 |
| 10 | newly 3 weeks spec | 159. | 307.86 | {13., 1294.} | 210.918 | 0.75246 | newly 3 weeks cumul | 644. | 1356.28 | {201., 3672.} | 172.64 | 0.395644 | newly 5 weeks spec | 135. | 98.14 | {0., 290.} | 118.815 | 1.51757 | newly 5 weeks cumul | 960. | 1622.3 | {229., 5236.} | 156. | 0.487747 | newly 51 weeks cumul | 1852. | 1765.41 | {264., 5873.} | 113.394 | 0.638928 |
| 11 | newly 3 weeks spec | 170. | 179.57 | {5., 151.} | 151.041 | 0.982006 | newly 3 weeks cumul | 592. | 1227.03 | {83., 2118.} | 186.691 | 0.492761 | newly 5 weeks spec | 147. | 15.73 | {0., 35.} | 91.8299 | 1.89228 | newly 5 weeks cumul | 872. | 1296.83 | {89., 2141.} | 151.79 | 0.586281 | newly 56 weeks cumul | 1526. | 1304.77 | {89., 2142.} | 119.188 | 0.766526 |
| 12 | newly 3 weeks spec | 240. | 88.32 | {12., 125.} | 76.8917 | 0.749042 | newly 3 weeks cumul | 1103. | 504.3 | {61., 602.} | 76.1723 | 0.667554 | newly 5 weeks spec | 100. | 33.78 | {3., 58.} | 77.78 | 0.833245 | newly 5 weeks cumul | 1384. | 590.25 | {90., 689.} | 76.1279 | 0.681377 | newly 58 weeks cumul | 1560. | 663.78 | {119., 780.} | 74.5372 | 0.664909 |
| 13 | newly 3 weeks spec | 357. | 174.03 | {2., 425.} | 91.1457 | 1.1156 | newly 3 weeks cumul | 1370. | 1812.39 | {552., 4348.} | 104.183 | 0.366526 | newly 5 weeks spec | 224. | 65.43 | {0., 47.} | 110.853 | 2.16302 | newly 5 weeks cumul | 1865. | 1980.24 | {558., 4667.} | 95.9678 | 0.43353 | newly 50 weeks cumul | 2415. | 2049.85 | {558., 4668.} | 86.8418 | 0.485819 |
| 14 | newly 3 weeks spec | 307. | 248.9 | {4., 845.} | 113.251 | 0.963918 | newly 3 weeks cumul | 1011. | 1603.27 | {149., 4283.} | 134.361 | 0.447294 | newly 5 weeks spec | 225. | 73.86 | {0., 253.} | 88.3467 | 1.70119 | newly 5 weeks cumul | 1501. | 1809.81 | {241., 4955.} | 116.654 | 0.518279 | newly 51 weeks cumul | 2614. | 1953.8 | {241., 5213.} | 90.3527 | 0.629056 |
| 15 | newly 3 weeks spec | 226. | 105.82 | {0., 166.} | 113.566 | 1.95817 | newly 3 weeks cumul | 696. | 1056.79 | {61., 2341.} | 136.875 | 0.522025 | newly 5 weeks spec | 226. | 11.14 | {0., 25.} | 95.0708 | 2.76305 | newly 5 weeks cumul | 1140. | 1102.7 | {61., 2358.} | 107.763 | 0.636727 | newly 53 weeks cumul | 3240. | 1108.48 | {61., 2358.} | 87.1037 | 0.97091 |
| 16 | newly 3 weeks spec | 319. | 184.76 | {25., 191.} | 103.53 | 0.75402 | newly 3 weeks cumul | 1150. | 906.85 | {147., 676.} | 106.749 | 0.563483 | newly 5 weeks spec | 224. | 65.3 | {2., 97.} | 89.6429 | 1.12171 | newly 5 weeks cumul | 1670. | 1079.27 | {202., 929.} | 101.271 | 0.625895 | newly 54 weeks cumul | 2574. | 1256.3 | {266., 1546.} | 92.7988 | 0.704151 |
| 17 | newly 3 weeks spec | 105. | 232.38 | {7., 681.} | 225.695 | 0.782734 | newly 3 weeks cumul | 312. | 2009.15 | {469., 6019.} | 545.843 | 0.532808 | newly 5 weeks spec | 82. | 37.02 | {0., 116.} | 94.9268 | 1.6446 | newly 5 weeks cumul | 477. | 2134.86 | {478., 6288.} | 350.998 | 0.377906 | newly 51 weeks cumul | 3040. | 2161.45 | {478., 6313.} | 85.7385 | 0.610736 |
| 18 | newly 3 weeks spec | 181. | 231.52 | {7., 834.} | 155.514 | 0.786639 | newly 3 weeks cumul | 580. | 893.28 | {53., 2336.} | 154.928 | 0.59479 | newly 5 weeks spec | 194. | 145.71 | {2., 239.} | 131.036 | 1.17651 | newly 5 weeks cumul | 959. | 1234.73 | {66., 5363.} | 147.863 | 0.702847 | newly 51 weeks cumul | 1686. | 1608.37 | {68., 7484.} | 128.028 | 0.83458 |
| 19 | newly 3 weeks spec | 266. | 181.16 | {27., 138.} | 107.805 | 0.673323 | newly 3 weeks cumul | 957. | 647.67 | {159., 515.} | 96.7074 | 0.561721 | newly 5 weeks spec | 162. | 105.69 | {9., 98.} | 110.167 | 0.784028 | newly 5 weeks cumul | 1331. | 895.77 | {211., 689.} | 100.518 | 0.596525 | newly 54 weeks cumul | 1835. | 1299.38 | {295., 1580.} | 98.5896 | 0.594178 |
| 20 | newly 3 weeks spec | 319. | 195. | {0., 145.} | 122.796 | 1.56068 | newly 3 weeks cumul | 1048. | 1175.57 | {61., 5664.} | 135.675 | 0.709261 | newly 5 weeks spec | 244. | 90.79 | {0., 96.} | 106.43 | 1.82025 | newly 5 weeks cumul | 1558. | 1405.03 | {68., 6947.} | 123.718 | 0.785043 | newly 55 weeks cumul | 2688. | 1576.57 | {68., 6969.} | 104.294 | 0.915643 |
| 21 | newly 3 weeks spec | 299. | 106.21 | {0., 205.} | 104.057 | 2.00432 | newly 3 weeks cumul | 1149. | 1075.95 | {105., 1610.} | 113.144 | 0.653434 | newly 5 weeks spec | 209. | 43.62 | {0., 81.} | 97.3301 | 2.0422 | newly 5 weeks cumul | 1607. | 1186.55 | {138., 1665.} | 103.596 | 0.706739 | newly 55 weeks cumul | 2136. | 1289.97 | {166., 1706.} | 96.7308 | 0.745956 |
| 22 | newly 3 weeks spec | 242. | 229.32 | {2., 760.} | 121.628 | 1.02518 | newly 3 weeks cumul | 748. | 1796.15 | {84., 5117.} | 192.611 | 0.472984 | newly 5 weeks spec | 169. | 28.43 | {0., 93.} | 86.2544 | 1.80792 | newly 5 weeks cumul | 1116. | 1906.62 | {84., 5494.} | 149.405 | 0.53877 | newly 51 weeks cumul | 2163. | 1923.78 | {84., 5527.} | 99.8363 | 0.668904 |
| 23 | newly 3 weeks spec | 259. | 197.47 | {8., 261.} | 104.413 | 0.72474 | newly 3 weeks cumul | 963. | 1128.73 | {213., 1939.} | 113.771 | 0.460254 | newly 5 weeks spec | 207. | 108.23 | {0., 94.} | 113.184 | 1.38524 | newly 5 weeks cumul | 1381. | 1378.19 | {245., 4685.} | 109.825 | 0.536225 | newly 56 weeks cumul | 1616. | 1631.26 | {253., 5530.} | 113.571 | 0.564025 |
| 24 | newly 3 weeks spec | 173. | 43.59 | {0., 48.} | 105.116 | 2.56053 | newly 3 weeks cumul | 546. | 393.66 | {120., 555.} | 76.2784 | 0.441714 | newly 5 weeks spec | 105. | 17.91 | {0., 19.} | 101.171 | 2.5809 | newly 5 weeks cumul | 774. | 438.64 | {122., 563.} | 83.4186 | 0.570671 | newly 52 weeks cumul | 1235. | 473.52 | {122., 563.} | 87.2891 | 0.75113 |
| 25 | newly 3 weeks spec | 260. | 191.54 | {1., 683.} | 125.569 | 1.33324 | newly 3 weeks cumul | 915. | 1368.08 | {31., 7601.} | 169.15 | 0.775681 | newly 5 weeks spec | 241. | 49.74 | {0., 37.} | 101.519 | 2.19474 | newly 5 weeks cumul | 1474. | 1504.7 | {32., 7800.} | 136.064 | 0.887504 | newly 59 weeks cumul | 2031. | 1616.13 | {34., 7808.} | 120.19 | 0.968755 |
| 26 | newly 3 weeks spec | 226. | 248.05 | {5., 1224.} | 150.677 | 0.972192 | newly 3 weeks cumul | 712. | 1316.48 | {63., 4547.} | 176.767 | 0.526755 | newly 5 weeks spec | 102. | 81.92 | {0., 127.} | 137.784 | 1.47542 | newly 5 weeks cumul | 971. | 1541.34 | {75., 6555.} | 165.668 | 0.60299 | newly 50 weeks cumul | 1344. | 1642.22 | {79., 8507.} | 143.902 | 0.691483 |
| 27 | newly 3 weeks spec | 316. | 56.5 | {0., 39.} | 103.399 | 2.36178 | newly 3 weeks cumul | 1146. | 800.93 | {36., 1182.} | 89.8578 | 0.674673 | newly 5 weeks spec | 192. | 5.63 | {0., 3.} | 98.0781 | 2.92756 | newly 5 weeks cumul | 1600. | 825.77 | {36., 1222.} | 88.1019 | 0.788005 | newly 52 weeks cumul | 1907. | 827.87 | {36., 1230.} | 86.8469 | 0.848723 |
| 28 | newly 3 weeks spec | 99. | 239.56 | {2., 769.} | 222.384 | 0.816348 | newly 3 weeks cumul | 324. | 1476.2 | {74., 3703.} | 382.846 | 0.610451 | newly 5 weeks spec | 91. | 118.69 | {0., 340.} | 143.33 | 1.18564 | newly 5 weeks cumul | 522. | 1762.79 | {101., 5670.} | 265.845 | 0.504267 | newly 30 weeks cumul | 1626. | 2112.19 | {166., 8191.} | 97.6144 | 0.392294 |
| 29 | newly 3 weeks spec | 301. | 29.54 | {0., 66.} | 93.9801 | 2.25919 | newly 3 weeks cumul | 953. | 831.53 | {126., 1529.} | 86.532 | 0.466283 | newly 5 weeks spec | 234. | 1.95 | {0., 6.} | 99.1667 | 2.90187 | newly 5 weeks cumul | 1506. | 840.6 | {126., 1529.} | 81.7955 | 0.608637 | newly 53 weeks cumul | 2280. | 841.38 | {126., 1529.} | 82.807 | 0.760743 |
| 30 | newly 3 weeks spec | 342. | 129.57 | {8., 166.} | 93.3947 | 0.954217 | newly 3 weeks cumul | 1048. | 785.37 | {93., 782.} | 95.9723 | 0.580669 | newly 5 weeks spec | 206. | 49.15 | {0., 88.} | 91.7233 | 1.49433 | newly 5 weeks cumul | 1528. | 909.2 | {109., 873.} | 93.4764 | 0.669081 | newly 52 weeks cumul | 2105. | 1066.56 | {114., 1640.} | 90.6347 | 0.73255 |
| 31 | newly 3 weeks spec | 270. | 167.86 | {0., 658.} | 113.541 | 1.37386 | newly 3 weeks cumul | 910. | 1329.87 | {27., 6502.} | 168.298 | 0.799982 | newly 5 weeks spec | 207. | 48.34 | {0., 49.} | 103.353 | 2.0691 | newly 5 weeks cumul | 1305. | 1452.41 | {31., 6952.} | 142.448 | 0.874606 | newly 59 weeks cumul | 1766. | 1585.65 | {31., 7852.} | 127.102 | 0.944915 |
| 32 | newly 3 weeks spec | 121. | 133.96 | {0., 606.} | 166.347 | 1.56516 | newly 3 weeks cumul | 377. | 1503.34 | {141., 8442.} | 377.645 | 0.476923 | newly 5 weeks spec | 116. | 17.64 | {0., 33.} | 99.3276 | 2.44937 | newly 5 weeks cumul | 611. | 1565.06 | {143., 9006.} | 265.119 | 0.604372 | newly 32 weeks cumul | 2466. | 1583.27 | {144., 9037.} | 115.004 | 0.992311 |
| 33 | newly 3 weeks spec | 364. | 36.42 | {0., 74.} | 95.2912 | 2.25759 | newly 3 weeks cumul | 1353. | 892.86 | {209., 1312.} | 70.9017 | 0.442173 | newly 5 weeks spec | 266. | 5.03 | {0., 8.} | 98.5677 | 2.93553 | newly 5 weeks cumul | 1933. | 911.14 | {209., 1312.} | 74.9457 | 0.568066 | newly 53 weeks cumul | 2442. | 913.79 | {209., 1312.} | 77.2699 | 0.654871 |
| 34 | newly 3 weeks spec | 262. | 39.07 | {0., 64.} | 98.3168 | 2.06125 | newly 3 weeks cumul | 877. | 818.47 | {90., 1686.} | 98.4823 | 0.530215 | newly 5 weeks spec | 247. | 2.88 | {0., 7.} | 98.834 | 2.93043 | newly 5 weeks cumul | 1378. | 831.36 | {91., 1686.} | 88.7721 | 0.662034 | newly 53 weeks cumul | 2645. | 834.04 | {91., 1686.} | 85.9191 | 0.891224 |
| 35 | newly 3 weeks spec | 285. | 95.82 | {0., 97.} | 103.789 | 2.03935 | newly 3 weeks cumul | 970. | 1285.76 | {176., 2523.} | 115.971 | 0.426106 | newly 5 weeks spec | 184. | 8.69 | {0., 14.} | 95.6902 | 2.53685 | newly 5 weeks cumul | 1379. | 1324.82 | {176., 2537.} | 102.841 | 0.52387 | newly 53 weeks cumul | 2032. | 1328.01 | {176., 2537.} | 93.6088 | 0.647459 |
| 36 | newly 3 weeks spec | 365. | 136.26 | {0., 85.} | 113.83 | 2.04584 | newly 3 weeks cumul | 1132. | 1176.12 | {27., 5513.} | 142.014 | 0.889084 | newly 5 weeks spec | 206. | 53.88 | {0., 49.} | 104.223 | 2.00985 | newly 5 weeks cumul | 1604. | 1318.25 | {27., 6929.} | 127.777 | 0.945215 | newly 55 weeks cumul | 2018. | 1414.92 | {32., 6929.} | 118.047 | 0.967347 |
| 37 | newly 3 weeks spec | 233. | 88.98 | {11., 108.} | 86. | 0.799479 | newly 3 weeks cumul | 842. | 505.95 | {59., 523.} | 85.5974 | 0.615843 | newly 5 weeks spec | 124. | 37.94 | {3., 58.} | 92.2903 | 1.02401 | newly 5 weeks cumul | 1099. | 601.19 | {82., 614.} | 86.0919 | 0.643791 | newly 58 weeks cumul | 1455. | 684.83 | {101., 740.} | 85.0289 | 0.686824 |
| 38 | newly 3 weeks spec | 329. | 147.18 | {32., 166.} | 89.8845 | 0.686067 | newly 3 weeks cumul | 996. | 543.71 | {219., 642.} | 79.8323 | 0.514343 | newly 5 weeks spec | 222. | 83.13 | {5., 93.} | 93.5631 | 0.87656 | newly 5 weeks cumul | 1520. | 735.85 | {285., 873.} | 83.5322 | 0.582703 | newly 54 weeks cumul | 2847. | 1043.45 | {320., 1329.} | 87.0604 | 0.721255 |
| 39 | newly 3 weeks spec | 207. | 103.66 | {1., 90.} | 108.541 | 1.11303 | newly 3 weeks cumul | 1062. | 913.16 | {98., 612.} | 121.041 | 0.68379 | newly 5 weeks spec | 83. | 35.03 | {0., 56.} | 102.47 | 1.43748 | newly 5 weeks cumul | 1296. | 1010.08 | {116., 642.} | 116.336 | 0.703953 | newly 55 weeks cumul | 1395. | 1077.55 | {126., 874.} | 113.318 | 0.690186 |
| 40 | newly 3 weeks spec | 80. | 33.12 | {1., 84.} | 87.3 | 0.971539 | newly 3 weeks cumul | 250. | 129.5 | {50., 285.} | 71.12 | 0.493633 | newly 5 weeks spec | 81. | 27.37 | {0., 107.} | 97.2716 | 1.66934 | newly 5 weeks cumul | 408. | 187.33 | {54., 440.} | 80.4779 | 0.633669 | newly 28 weeks cumul | 1434. | 283.21 | {54., 1092.} | 84.2155 | 1.05654 |
| 41 | newly 3 weeks spec | 318. | 153.1 | {0., 538.} | 105.899 | 1.39361 | newly 3 weeks cumul | 1244. | 1235.95 | {46., 5938.} | 137.768 | 0.882875 | newly 5 weeks spec | 73. | 38.02 | {0., 50.} | 108.74 | 1.60306 | newly 5 weeks cumul | 1452. | 1346.42 | {47., 6364.} | 131.532 | 0.896344 | newly 59 weeks cumul | 1481. | 1473.76 | {47., 6376.} | 134.752 | 0.891715 |
| 42 | newly 3 weeks spec | 264. | 196.32 | {5., 540.} | 102.129 | 0.892844 | newly 3 weeks cumul | 803. | 664.77 | {44., 1729.} | 96.3873 | 0.607009 | newly 5 weeks spec | 207. | 143.25 | {0., 472.} | 110.082 | 1.19746 | newly 5 weeks cumul | 1233. | 975.88 | {51., 2721.} | 98.4169 | 0.680398 | newly 51 weeks cumul | 1934. | 1593.19 | {73., 5488.} | 108.219 | 0.772091 |
| 43 | newly 3 weeks spec | 267. | 40.86 | {0., 72.} | 90.7191 | 1.90583 | newly 3 weeks cumul | 1014. | 1049.62 | {99., 2451.} | 135.949 | 0.700987 | newly 5 weeks spec | 120. | 27.92 | {0., 55.} | 91.5667 | 1.96236 | newly 5 weeks cumul | 1409. | 1108.83 | {102., 2564.} | 117.359 | 0.746204 | newly 57 weeks cumul | 1476. | 1254.71 | {113., 2564.} | 113.246 | 0.676799 |
| 44 | newly 3 weeks spec | 266. | 103.49 | {0., 169.} | 96.4098 | 1.37424 | newly 3 weeks cumul | 845. | 544.02 | {63., 836.} | 88.7361 | 0.643203 | newly 5 weeks spec | 232. | 71.85 | {0., 93.} | 101.099 | 1.84871 | newly 5 weeks cumul | 1322. | 700.1 | {86., 1006.} | 89.4493 | 0.738925 | newly 53 weeks cumul | 2625. | 1058.43 | {104., 1292.} | 92.9398 | 0.930469 |
| 45 | newly 3 weeks spec | 490. | 384.1 | {2., 2599.} | 135.09 | 1.45525 | newly 3 weeks cumul | 1749. | 1009.49 | {21., 5008.} | 98.8754 | 0.975318 | newly 5 weeks spec | 208. | 120.47 | {0., 675.} | 118.813 | 1.91093 | newly 5 weeks cumul | 2301. | 1379.96 | {23., 7567.} | 103.272 | 1.04244 | newly 60 weeks cumul | 2545. | 1504.45 | {23., 8105.} | 102.919 | 1.06281 |
| 46 | newly 3 weeks spec | 320. | 57.36 | {0., 92.} | 89.8313 | 1.6435 | newly 3 weeks cumul | 1046. | 1376.81 | {99., 3179.} | 155.36 | 0.686908 | newly 5 weeks spec | 217. | 26.72 | {0., 64.} | 92.1935 | 2.19827 | newly 5 weeks cumul | 1558. | 1435.88 | {99., 3199.} | 125.729 | 0.754282 | newly 57 weeks cumul | 1999. | 1638.56 | {99., 5844.} | 113.687 | 0.761737 |
| 47 | newly 3 weeks spec | 449. | 39.71 | {0., 88.} | 93.4143 | 2.07288 | newly 3 weeks cumul | 1325. | 1200.38 | {58., 2219.} | 129.645 | 0.832941 | newly 5 weeks spec | 304. | 25.5 | {0., 54.} | 96.4737 | 2.42919 | newly 5 weeks cumul | 2024. | 1254.21 | {67., 2277.} | 108.744 | 0.908379 | newly 57 weeks cumul | 2952. | 1383.96 | {95., 2277.} | 99.0346 | 0.944116 |
| 48 | newly 3 weeks spec | 220. | 141.67 | {45., 145.} | 87.7955 | 0.508685 | newly 3 weeks cumul | 864. | 521.78 | {224., 570.} | 76.6204 | 0.439344 | newly 5 weeks spec | 200. | 83.67 | {12., 84.} | 92.725 | 0.791026 | newly 5 weeks cumul | 1267. | 717.46 | {281., 740.} | 80.8745 | 0.492618 | newly 54 weeks cumul | 1825. | 1047.38 | {327., 1097.} | 86.6751 | 0.549468 |
| 49 | newly 3 weeks spec | 192. | 169.53 | {0., 303.} | 124.505 | 1.07565 | newly 3 weeks cumul | 594. | 2129.41 | {70., 8766.} | 302.995 | 0.536133 | newly 5 weeks spec | 164. | 13.03 | {0., 33.} | 93.7866 | 2.25491 | newly 5 weeks cumul | 949. | 2189.74 | {74., 9178.} | 188.516 | 0.500557 | newly 50 weeks cumul | 2640. | 2196.73 | {74., 9202.} | 105.378 | 0.745826 |
| 50 | newly 3 weeks spec | 145. | 226.96 | {19., 632.} | 153.338 | 0.568342 | newly 3 weeks cumul | 506. | 1620.87 | {259., 7250.} | 266.883 | 0.338062 | newly 5 weeks spec | 93. | 73.2 | {1., 54.} | 122.817 | 0.985675 | newly 5 weeks cumul | 715. | 1817.05 | {272., 8573.} | 221.917 | 0.398266 | newly 50 weeks cumul | 1140. | 1942.19 | {273., 9338.} | 168.111 | 0.508386 |
